# Downregulation of proapoptotic Bim augments IL-2-independent T-cell transformation by human T-cell leukemia virus type-1 Tax

**DOI:** 10.1002/cam4.329

**Published:** 2014-08-30

**Authors:** Masaya Higuchi, Masahiko Takahashi, Yuetsu Tanaka, Masahiro Fujii

**Affiliations:** 1 Division of Virology, Niigata University Graduate School of Medical and Dental SciencesNiigata, Japan; 2 Department of Immunology, Graduate School and Faculty of Medicine, University of the RyukyusOkinawa, Japan

**Keywords:** ATL, apoptosis, BIM protein, ERK MAP kinases, HTLV-1 Tax genes

## Abstract

Human T-cell leukemia virus type 1 (HTLV-1), an etiological agent of adult T-cell leukemia, immortalizes and transforms primary human T cells in vitro in both an interleukin (IL)-2-dependent and IL-2-independent manner. Expression of the HTLV-1 oncoprotein Tax transforms the growth of the mouse T-cell line CTLL-2 from being IL-2-dependent to IL-2-independent. Withdrawal of IL-2 from normal activated T cells induces apoptosis, which is mediated through the inducible expression of several proapoptotic proteins, including Bim. In this study, we found that Tax protects IL-2-depleted T cells against Bim-induced apoptosis. Withdrawal of IL-2 from CTLL-2 cells induced a prominent increase in the level of Bim protein in CTLL-2 cells, but not in Tax-transformed CTLL-2 cells. This inhibition of Bim in Tax-transformed CTLL-2 cells was mediated by two mechanisms: downregulation of *Bim* mRNA and posttranscriptional reduction of Bim protein. Transient expression of Tax in CTLL-2 cells also inhibited IL-2 depletion–induced expression of Bim, however, this decrease in Bim protein expression was not due to downregulation of *Bim* mRNA, thus indicating that *Bim* mRNA downregulation in Tax-transformed CTLL-2 occurs only after long-term expression of Tax. Transient expression of Tax in CTLL-2 cells also induced Erk activation, however, this was not involved in the reduction of Bim protein. Knockdown of Bim expression in CTLL-2 cells augmented Tax-induced IL-2-independent transformation. HTLV-1 infection of human T cells also reduced their levels of Bim protein, and restoring Bim expression in HTLV-1-infected cells reduced their proliferation by inducing apoptosis. Taken together, these results indicate that Tax-induced downregulation of Bim in HTLV-1-infected T cells promotes their IL-2-independent growth, thereby supporting the persistence of HTLV-1 infection in vivo.

## Introduction

Adult T-cell leukemia (ATL) is a highly aggressive human leukemia characterized by the clonal proliferation of mature T cells and is initiated by an infection with human T-cell leukemia virus type 1 (HTLV-1) [1, 2]. HTLV-1 transmission mainly occurs from mothers to their infants through breast milk. Approximately 3–5% of HTLV-1 infections develop into ATL, at an average age of 60 years [3]. During asymptomatic periods, host immunity suppresses the outgrowth of HTLV-1-infected cells. However, two events, deterioration of host immunity and emergence of a malignant clone with genetic and epigenetic alterations, overcome suppression against HTLV-1-infected T cells, resulting in the development of ATL [4, 5].

HTLV-1 immortalizes primary human T cells in vitro, and subsequently some cells acquire an interleukin-2 (IL-2)-independent growth phenotype [6]. This HTLV-1-induced immortalization and transformation event is essential for establishing a lifelong persistent infection. In addition to structural genes, HTLV-1 encodes two oncoproteins, Tax and HBZ [1]. Of the two, Tax is essential for HTLV-1-mediated IL-2-dependent immortalization of T cells. For example, mutation of the *tax* gene in a recombinant HTLV-1 strain abolishes its immortalization activity in T cells [7]. Moreover, Tax alone, without any other viral genes, can immortalize T cells in vitro [8, 9]. In addition to IL-2-dependent immortalization, Tax may also play a role in the IL-2-independent transformation of T cells by HTLV-1. For instance, transduction of the *tax* gene into the mouse IL-2-dependent T-cell line CTLL-2 confers IL-2-independent growth [10]. Tax has been reported to repress the proapoptotic Bcl-2 family protein Bax and induce the antiapoptotic proteins Bcl-xL and Bfl-1 [11–13]. However, how Tax induces the IL-2-independent growth transformation in T cells has not been fully elucidated.

Upon depletion of IL-2, activated normal T cells initiate apoptosis through the induction of several proapoptotic genes, including *Bim* and *Fas* ligand [14]. Bim is a proapoptotic BH3-only protein, which binds to all members of the antiapoptotic Bcl-2 family [15]. In this study, we examined how Tax prevents Bim-induced apoptosis of T cells after IL-2 depletion. We present evidence that downregulation of Bim in T cells plays a crucial role in the IL-2-independent growth of HTLV-1-infected T cells, including ATL-derived cells.

## Materials and Methods

### Cells and cell culture conditions

CTLL-2 is a mouse T-cell line that grows in an IL-2-dependent manner. CTLL-2/Tax is a Tax-transformed CTLL-2 cell line that grows in an IL-2-independent manner [16]. CTLL-2 cells were cultured in RPMI 1640 medium supplemented with 10% fetal bovine serum (FBS) and 55 *μ*mol/L 2-mercaptoethanol (2-ME) (RPMI/10% FBS) plus 0.5 nmol/L recombinant human IL-2 (Takeda Pharmaceutical Company, Osaka, Japan). CTLL-2/Tax cells were cultured in RPMI/10% FBS. The human T-cell lines used in the present study have been characterized previously [10] and include Jurkat and MOLT-4, which are HTLV-1-negative; C5/MJ, C91/PL, HUT-102, MT-2, MT-4, and SLB-1, which are HTLV-1-transformed; and KK1 [17], KOB [18], ST1 [19], MT-1, and TL-OmI, which were derived from ATL patients. These cells were cultured in RPMI/10% FBS. Human IL-2 was added to a final concentration of 0.5 nmol/L to the culture of KK1, KOB, and ST1 cells. Human peripheral blood mononuclear cells (PBMC) were isolated from the blood of a healthy donor using Ficoll-Paque PLUS (GE Healthcare, Uppsala, Sweden). PBMCs were stimulated with 10 *μ*g/mL phytohemagglutinin (PHA) (Sigma-Aldrich, St. Louis, MO) in RPMI 1640 medium supplemented with 20% FBS and 55 *μ*mol/L 2-ME (RPMI/20% FBS) for 2 days and then further cultured in RPMI/20% FBS plus 0.5 nmol/L IL-2. 293T is a human embryonic kidney cell line and was cultured in Dulbecco's modified Eagle's medium supplemented with 10% FBS. To inhibit MEK activity, CTLL-2 cells were treated with 1, 3, and 10 *μ*mol/L U0126 (Merck Millipore, Billerica, MA) for 18 h.

### Plasmids

CSII-EF-IB, a lentiviral expression vector coexpressing the blasticidin resistance gene, was used for expressing Tax, Tax mutants, and enhanced green fluorescent protein (EGFP) as a control [20]. To make the expression vectors, Tax, Tax225-232 (defective for NF-*κ*B2 activation) [21], TaxΔC (defective for PDZ domain protein binding) [22], Tax703 (defective for CREB activation), and TaxM22 (defective for NF-*κ*B activation) [23] genes were amplified by polymerase chain reaction (PCR), cloned into pENTR/D-TOPO (Life Technologies, Carlsbad, CA), and then transferred to the CSII-EF-IB vector using LR Clonase (Life Technologies). CSII-EF-EGFP and CSII-EF-Tax1 are the lentiviral expression vectors separately encoding EGFP and Tax1, respectively [24]. Human BimEL cDNA was amplified by PCR, cloned into pENTR/D-TOPO, and transferred to CSII-EF-IG, a lentiviral bicistronic EGFP expression vector [20], by an LR reaction. Lentiviral vectors expressing short hairpin RNAs (shRNAs) against mouse *Bim* mRNA or control shRNA were purchased from Sigma.

### Lentiviruses

Recombinant lentiviruses were generated by transfecting each lentiviral vector together with pCAG-HIVgp and pCMV-VSV-G-RSV-Rev (provided by Dr. H. Miyoshi, RIKEN Tsukuba Institute) into 293T cells by lipofection using FuGENE HD (Promega, Madison, WI). Since transfection of the BimEL-expressing lentiviral vector into 293T cells induced cell death, the pSVBT plasmid expressing the human antiapoptotic protein Bcl-2 (provided by Dr. Y. Tsujimoto at Osaka University) was cotransfected into 293T cells. The supernatant of 293T cells containing the lentiviruses was used to infect CTLL-2, TL-OmI, and ST1 cells (2–4 × 10^5^) in a final volume of 1 mL of RPMI/10% FBS containing 8 *μ*g/mL polybrene, supplemented with 0.5 nmol/L IL-2 for CTLL-2 cells. The efficiency of these infections was augmented by centrifugation of the infected cells with the viruses (800*g* at 32°C for 1 h) as described previously [25]. To establish stably infected CTLL-2 cell lines, infected cells were cultured in selection medium containing 28 *μ*g/mL blasticidin or 2 *μ*g/mL puromycin for 1–2 weeks.

### Western blotting

Total cell extracts were prepared by lysing cells in SDS sample buffer (2% SDS, 62.5 mmol/L Tris-HCl [pH 6.8], 10% glycerol, 50 mmol/L dithiothreitol, and 0.01% bromophenol blue) and heating the mixtures at 95°C for 5 min. The proteins in the extracts were separated by SDS polyacrylamide gel electrophoresis, transferred onto a polyvinylidene difluoride (PVDF) membrane (Bio-Rad Laboratories, Hercules, CA), and probed with anti-Tax (Taxy7) [26], p-Erk (Santa Cruz Biotechnology, Dallas, TX), Bim, *α*-Tubulin (Merck Millipore), Bax, Erk1/2 (Cell Signaling Technology, Danvers, MA), or Bcl-xL antibodies (BD Biosciences, San Jose, CA). The intensity of bands was measured using ImageJ software.

### Real-time PCR

Total RNA was isolated from CTLL-2 or CTLL-2/Tax cells using the RNAiso reagent (Takara Bio, Otsu, Japan). Five hundred nanograms of total RNA was reverse-transcribed using the ExScript RT reagent kit (Takara Bio). To quantify the amount of *Bim* RNA, real-time PCR based on SYBR green fluorescence was performed using SYBR Premix Ex Taq polymerase and the Thermal Cycler Dice real-time system (Takara Bio). Primers specific for mouse *Bim* and glyceraldehyde-3-phosphate dehydrogenase (*GAPDH*) RNAs were purchased from Takara Bio.

### Transformation assay

The IL-2-independent transformation assay was performed as described previously [16]. Briefly, CTLL-2 cells were infected with the Tax or Tax225-232 lentivirus, and the cells were cultured in 96-well plates (0.1 × 10^4^, 0.3 × 10^4^, and 1 × 10^4^ cells per well) without IL-2 for 4 weeks. The number of wells containing outgrowing cells was counted under a light microscope.

### Cell death assays

Cell viability was assessed by staining with trypan blue under a light microscope. To monitor for apoptosis, cells were stained with 5 *μ*g/mL of Hoechst 33342 for 30 min at 37°C and examined with an inverted fluorescence microscope (Keyence, Osaka, Japan).

### Statistical analysis

Statistical analysis was performed using GraphPad Prism 5 software (GraphPad Software Inc., San Diego, CA).

## Results

### Bim downregulation in Tax-transformed CTLL-2 cells

**Figure 1:**
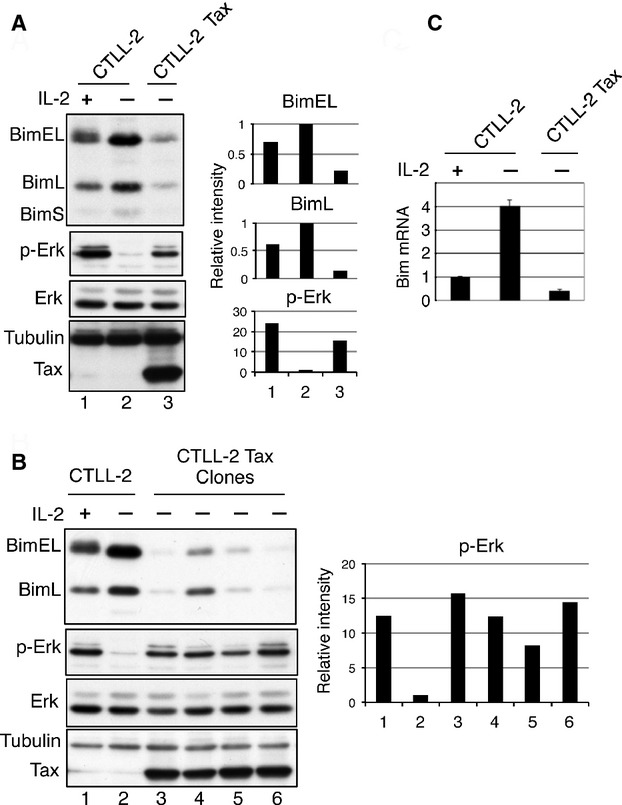
Downregulation of Bim in Tax-transformed CTLL-2 cells. (A, B) Cell lysates were prepared from CTLL-2 cells cultured in the presence or absence of IL-2 for 18 h and from Tax-transformed CTLL-2 cells cultured without IL-2, and the levels of Bim, p-Erk, Tax, and *α*-tubulin were analyzed by western blotting using the corresponding antibodies. Band intensities of Bim and p-Erk were quantified and normalized to *α*-tubulin and Erk, respectively. (C) Total RNA was prepared from CTLL-2 cells cultured in the presence or absence of IL-2 for 18 h and from Tax-transformed CTLL-2 cells cultured without IL-2. The amount of *Bim* transcript was measured by real-time PCR and normalized to the amount of *GAPDH* RNA.

CTLL-2 is a mouse T-cell line that requires IL-2 for continuous growth. Since depletion of IL-2 from CTLL-2 cultures induces apoptosis, we first examined whether IL-2 withdrawal from CTLL-2 cells induces the expression of the proapoptotic protein, Bim. Western blot analysis showed that IL-2 withdrawal from CTLL-2 cells for 18 h markedly induced the expression of BimEL, BimL, and BimS (Fig. 1A), which are proteins encoded by the three alternatively spliced variants of the identical *Bim* transcript. All three isoforms have a proapoptotic function, with BimS being the most potent [27]. This observation suggests that Bim is one factor responsible for IL-2 depletion–induced apoptosis of CTLL-2 cells.

We have previously shown that Tax transforms the growth of CTLL-2 cells from being IL-2-dependent to IL-2-independent [10]. Therefore, we next examined how Tax inactivates Bim in IL-2-depleted CTLL-2 cells. We found that the amount of Bim proteins in Tax-transformed CTLL-2 cells in the absence of IL-2 was much lower than that in IL-2-depleted parental CTLL-2 cells, and was even lower than that in IL-2-supplemented CTLL-2 cells (Fig. 1A). In addition, four independently established Tax-transformed IL-2-independent CTLL-2 clones displayed a reduced amount of Bim protein in the absence of IL-2 (Fig. 1B). These results suggest that Tax prevents IL-2 depletion–induced apoptosis of CTLL-2 cells partly through reducing the level of Bim protein.

The amount of Bim protein in lymphocytes is regulated at transcriptional, posttranscriptional and posttranslational levels [28–33]. At the posttranslational level, Bim is phosphorylated at three serine residues by Erk kinase and is subsequently degraded through the ubiquitin-mediated proteasome-dependent pathway [34]. As expected, the active, phosphorylated form of Erk (p-Erk) was present in IL-2-supplemented CTLL-2 cells, and the amount of p-Erk was decreased by IL-2 depletion (Fig. 1A). In contrast, the amount of p-Erk in Tax-transformed cells, including the four CTLL-2 clones (CTLL-2/Tax), was much higher than that in IL-2-depleted CTLL-2 cells (Fig. 1A and B), suggesting that Tax activates Erk in IL-2-depleted CTLL-2 cells.

We next examined whether Tax also represses Bim at the RNA level in CTLL-2 cells. Real-time PCR analysis showed that *Bim* mRNA in CTLL-2 cells was greatly induced by IL-2 deprivation, but no induction of *Bim* mRNA was observed in Tax-transformed CTLL-2 cells cultured in the absence of IL-2 (Fig. 1C). These results suggest that Tax-induced IL-2-independent transformation of CTLL-2 cells is due to downregulation of Bim at both the protein and the mRNA levels.

**Figure 2:**
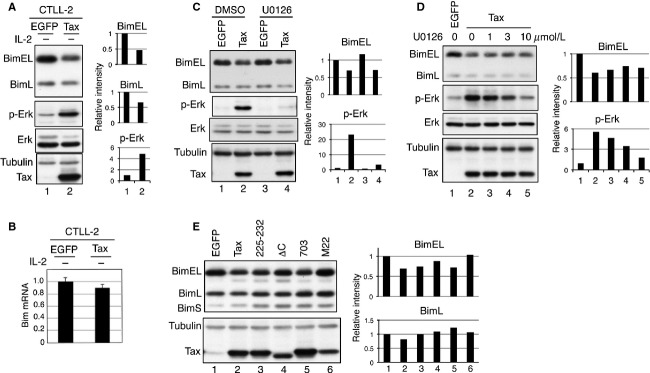
Transient expression of Tax induces Bim protein degradation in CTLL-2 cells. (A) CTLL-2 cells were infected with lentivirus expressing Tax or control EGFP. Total cell lysates were prepared from the infected cells cultured in the absence of IL-2 for 18 h, and the levels of Bim, p-Erk, Tax, and *α*-tubulin were analyzed by western blotting using the corresponding antibodies. (B) CTLL-2 cells infected with Tax- or EGFP-expressing lentiviruses were cultured without IL-2 for 18 h. The amount of *Bim* transcript in these cells was measured by real-time PCR analysis and normalized to the amount of *GAPDH* RNA. (C) CTLL-2 cells were infected with the lentivirus coexpressing Tax and blasticidin-S-deaminase, selected with blasticidin for 7 days, and cultured without IL-2 for 18 h in the absence (DMSO) or presence of 10 *μ*mol/L U0126. Total cell lysates were prepared and the expression of Bim, p-Erk, Tax, and *α*-tubulin proteins was determined by western blot analysis. (D) CTLL-2 cells were infected as in (C) and cultured without IL-2 for 18 h in the absence or presence of 1, 3, and 10 *μ*mol/L U0126. Total cell lysates were prepared and the expression of Bim, p-Erk, Tax, and *α*-tubulin proteins was determined by western blot analysis. (E) CTLL-2 cells were infected with the indicated lentiviruses, selected with blasticidin for 7 days and cultured without IL-2 for 18 h. Total cell lysates were prepared and the expression of Bim, p-Erk, Tax, and *α*-tubulin proteins was determined by western blot analysis. Band intensities of Bim and p-Erk were quantified and normalized to *α*-tubulin and Erk, respectively.

To further establish that Tax reduces the expression of Bim in CTLL-2 cells, we transiently expressed Tax in CTLL-2 cells by infection with a Tax-expressing lentivirus. The Tax virus–infected cells were cultured in the presence of IL-2 for 48 h and further cultured without IL-2 for 18 h. Transient expression of Tax in CTLL-2 cells reduced the amount of BimEL and BimL protein, with the reduction of BimEL being greater than that of BimL (Fig. 2A). It should be noted that BimEL, but not BimL, has three Erk phosphorylation sites, serines 55, 65, and 73, which control Erk-induced degradation of Bim [35]. The greater amount of p-Erk in Tax-expressing CTLL-2 cells than in control CTLL-2 cells and the slightly slower SDS-PAGE gel migration of BimEL, but not BimL, in Tax-expressing CTLL-2 cells (Fig. 2A) are consistent with Tax-induced Erk-dependent phosphorylation of BimEL. Collectively, these results suggest that transient expression of Tax activates Erk to phosphorylate BimEL, thereby inducing the degradation of BimEL. Consistent with this, transient expression of Tax in CTLL-2 cells minimally affected the level of *Bim* mRNA (Fig. 2B). Therefore, the observed downregulation of *Bim* mRNA in Tax-transformed IL-2-independent CTLL-2 cells is either a consequence of long-term expression of Tax or due to selection for Bim low-expressing cells during the Tax-induced IL-2-independent transformation process of CTLL-2 cells.

To further examine the finding that Tax reduces Bim protein level through Erk activation, CTLL-2 cells were infected with lentivirus coexpressing Tax and blasticidin-S-deaminase, and the cells were cultured with IL-2 and blasticidin for 7 days. These cells were treated with the MEK inhibitor U0126 in the absence of IL-2, and the amount of Bim in these cells was examined (Fig. 2C and D). While Tax reduced the amount of BimEL protein in CTLL-2 cells and U0126 almost completely blocked Erk activation by Tax, U0126 did not significantly affect the reduction of BimEL, indicating that Tax reduces Bim protein by an Erk-independent mechanism.

Next, we characterized various Tax mutants for their Bim-reducing activities (Fig. 2E). To do so, CTLL-2 cells were infected with lentivirus coexpressing Tax and blasticidin-S-deaminase, cultured with IL-2 and blasticidin for 7 days, and examined for Bim and p-Erk expression after IL-2 withdrawal for 18 h. Wild-type Tax (WT-Tax) reduced the expression of BimEL but not BimL in CTLL-2 cells. Among the Tax mutants, TaxΔC and M22 did not induce a reduction in Bim expression. Since TaxΔC lacks a PDZ domain–binding motif (PBM) [22] and M22 does not activate NF-*κ*B-dependent transcription [23], these results suggest that functions mediated by the PBM and NF-*κ*B signaling pathways are both required for Tax-induced Bim reduction. It should be noted, however, that the amount of TaxΔC and M22-mutant proteins in CTLL-2 cells was lower than that of WT-Tax. Thus, further analysis is required to confirm the above conclusion. In contrast, Tax703 and Tax225-232 reduced Bim expression equivalent to WT-Tax in CTLL-2 cells. Since Tax703 and Tax225-232 are defective for CREB activation [23] and NF-*κ*B2-dependent transcriptional activation [21], respectively, our results indicate that the activation of both CREB and NF-*κ*B2 pathways by Tax are dispensable for the Tax-induced reduction of Bim.

### Knockdown of Bim augments the transforming activity of Tax in CTLL-2 cells

**Figure 3:**
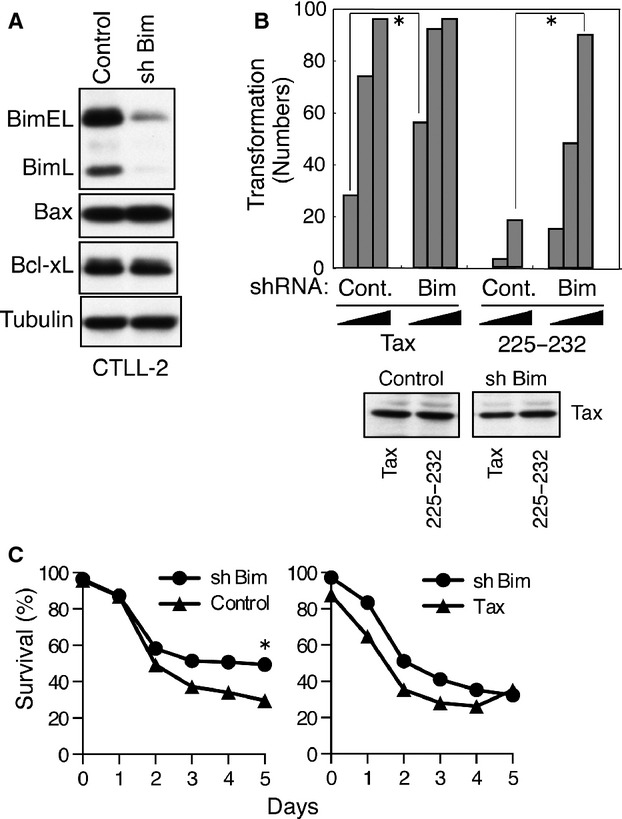
Knockdown of Bim augments the transforming activities of Tax. (A) CTLL-2 cells were infected with lentivirus encoding *Bim* shRNA or control shRNA, and the cells were cultured with puromycin for more than 1 week. Cell lysates were prepared from the selected cells after IL-2 withdrawal and the amount of Bim, Bax, Bcl-xL, and *α*-tubulin present was determined by western blot analysis. (B) Bim-knockdown and control CTLL-2 cells were infected with lentivirus expressing Tax or Tax225–232, and the cells were cultured in 96-well plates (0.1 × 10^4^, 0.3 × 10^4^, and 1 × 10^4^ cells per well) without IL-2 for 4 weeks. The number of wells containing outgrowing cells was determined using a light microscope (*P < 0.0001 by the chi-square test). Cell lysates were prepared 48 h after infection with the indicated viruses, and the amount of the Tax protein in the lysates was determined by western blot analysis. Data are representative of two independent experiments. (C) Bim-knockdown and control CTLL-2 cells (left) or Bim-knockdown and Tax-expressing CTLL-2 cells (right) were cultured without IL-2 for 5 days. The viability of the cells was evaluated by trypan blue staining using a light microscope (*P < 0.0001 by the *Z*-test). Data are representative of two independent experiments.

To investigate whether Bim downregulation by Tax is involved in the transformation of CTLL-2 cells, we established Bim-knockdown CTLL-2 cells by infection of lentivirus expressing shRNA targeting *Bim*. BimEL and BimL were efficiently knocked down in these cells, but the levels of other Bcl-2 family proteins, Bax and Bcl-xL, were not affected (Fig. 3A). Bim knockdown did not induce IL-2-independent growth transformation of CTLL-2 cells without Tax (data not shown). Next, Bim-knockdown and control CTLL-2 cells were infected with lentivirus encoding WT-Tax or a Tax mutant (Tax225-232) defective for NF-*κ*B2 activation, and the cells were cultured in the absence of IL-2 for 4 weeks. Tax efficiently induced IL-2-independent transformation in CTLL-2 cells, and the transforming activity was augmented by Bim knockdown (Fig. 3B). Consistent with our previous report [21], Tax225–232 had a lower transformation capacity than WT-Tax in the control cells, but its transforming activity in CTLL-2 was also greatly improved by Bim knockdown. The expression levels of WT and mutant Tax proteins 48 h after infection with their corresponding lentiviral vectors were almost equivalent (Fig. 3B). Taken together, these results indicate that Bim induction by IL-2 deprivation in CTLL-2 cells inhibits their transformation by WT-Tax and Tax225–232, and that Bim downregulation by Tax is an important activity for the cells' acquisition of IL-2 independence. In addition, these results suggest that the lower transforming activity of Tax225–232 relative to WT-Tax is mediated by the mechanism distinct from the reduction of Bim.

We next compared the role of Tax expression and Bim-knockdown in CTLL-2 cells during the initial phase of CTLL-2 cell transformation. CTLL-2 cells transduced with Tax-lentivirus in the presence of IL-2 and Bim-knockdown CTLL-2 cells were cultured in the absence of IL-2 (Fig. 3C, right). Tax expression in CTLL-2 cells did not confer a survival advantage compared with Bim-knockdown after 3 days, however, at day 4 Tax-expressing cells, but not Bim-knockdown cells, started to grow. These results suggest that BimEL downregulation by Tax in CTLL-2 is not enough to inhibit apoptosis of CTLL-2 after IL-2 withdrawal. Consistently, Bim knockdown only partially protected CTLL-2 cells from IL-2-depletion–induced apoptosis (Fig. 3C, left).

### Downregulation of Bim in HTLV-1-infected T-cell lines

**Figure 4:**
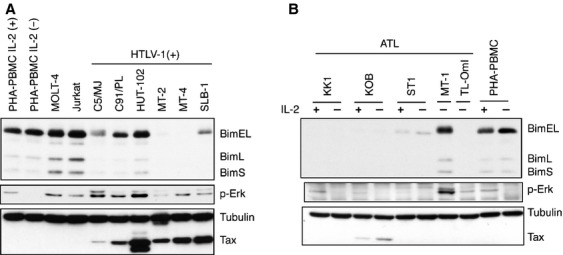
Expression of Bim in HTLV-1-infected T-cell lines. (A) Cell lysates were prepared from six HTLV-1-infected T-cell lines, two HTLV-1-uninfected T-cell lines and PHA-stimulated PBMC. The levels of Bim, p-Erk, Tax, and *α*-tubulin proteins were determined by western blot analysis using the corresponding antibodies. (B) Cell lysates were prepared from ATL-derived T-cell lines or PHA-PBMC cultured with or without IL-2 for 18 h. The levels of Bim, Tax, p-Erk, and *α*-tubulin proteins were determined by western blot analysis using the corresponding antibodies.

We next investigated the status of Bim protein in HTLV-1-transformed IL-2-independent T-cell lines and in T-cell lines that originated from ATL patients. Western blot analysis showed that five of the six HTLV-1-transformed T-cell lines (all except HUT102) expressed a reduced amount of Bim protein relative to uninfected T-cell lines and PHA-activated peripheral blood mononuclear cells (PHA-PBMC) (Fig. 4A). Since HTLV-1-transformed cells express less BimL and BimS than uninfected T-cell lines, the corresponding *Bim* mRNAs are presumed to be downregulated in these HTLV-1-transformed cell lines. These results suggest that downregulation of Bim protein is a factor required for HTLV-1-mediated IL-2-independent transformation of human T cells. In addition, four of the five ATL cell lines tested (all except MT-1) expressed a reduced amount of Bim protein relative to PHA-PBMC (Fig. 4B). It should be noted that MT-1 expressed a relatively high level of p-Erk. Furthermore, the BimEL detected in MT-1 cells had a slightly higher molecular weight than that in ST1 cells, suggesting that it may be inactivated by posttranslational modifications such as phosphorylation. Collectively, these results suggest that Bim is inactivated in ATL cells. Of the five ATL cell lines, only KOB cells expressed a detectable amount of Tax. Thus, the downregulation of Bim protein in ATL-derived cell lines is mostly Tax-independent, and another viral protein or a host factor appears to control the amount of Bim in ATL cells.

### Re-expression of Bim in ATL cell lines induces apoptosis

**Figure 5:**
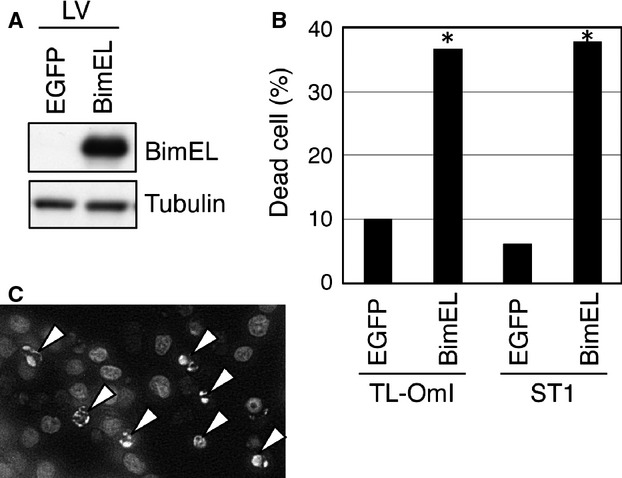
Re-expression of Bim in ATL cell lines induces apoptosis. (A, B, C) TL-OmI and ST1 cells were infected with lentivirus (LV) expressing Bim or control EGFP. Cell lysates were prepared from ST1 cells 24 h after infection, and the level of Bim protein was analyzed by western blotting (A). Cell viability 48 h after infection was evaluated by the trypan blue dye exclusion method (*P < 0.0001 by the *Z*-test) (B). Bim-transduced ST1 cells were evaluated for apoptosis by nuclear staining with Hoechst 33342. Arrows indicate the condensed nuclei of apoptotic cells (C). Data are representative of two independent experiments.

To establish whether the downregulation of Bim in ATL cell lines plays a role in their aberrant growth properties, we re-expressed Bim in Bim-negative ATL cell lines. TL-OmI and ST1 cells were infected with the EGFP- or BimEL-expressing lentivirus, and 48 h after infection cell viability was determined by trypan blue staining. BimEL was detected in BimEL-transduced ST1 cells but not in control cells (Fig. 5A). Furthermore, BimEL re-expression in the two ATL cell lines significantly induced cell death compared with control expression of EGFP (Fig. 5B). Hoechst 33342 staining revealed condensed chromatin in BimEL-expressing ST1 cells, indicating that the observed cell death was induced by an apoptotic mechanism (Fig. 5C). These results demonstrate that the downregulation of Bim in ATL cells is required for their aberrant cell growth and survival.

## Discussion

HTLV-1 immortalizes and transforms human T cells in both an IL-2-dependent and IL-2-independent manner in vitro, suggesting that HTLV-1-infected cells in vivo grow at both high-IL-2 and low-IL-2 conditions [36]. In this study, we found that Tax attenuates the induction of Bim protein in the T-cell line CTLL-2 after IL-2 depletion, and that this reduction promotes the Tax-induced IL-2-independent transformation of CTLL-2 cells. Moreover, HTLV-1-transformed IL-2-independent human T-cell lines generally displayed a low level of Bim protein relative to control T-cell lines. These results suggest that Tax-induced Bim reduction plays a role in cell growth in HTLV-1-infected T cells under IL-2-depleted or low-IL-2 conditions.

Reduction of Bim in Tax-transformed or Tax-expressing CTLL-2 cells seems to occur by two distinct mechanisms, one being the downregulation of *Bim* mRNA level. Since transient expression of Tax in CTLL-2 cells did not reduce Bim mRNA, there are two possible mechanisms to explain the downregulation of *Bim* mRNA in Tax-transformed cells. First, because Tax interacts with several chromatin remodeling factors, such as the histone methyltransferase SUV39H1 [37], Tax might induce the silencing of the *Bim* gene. Alternatively, only CTLL-2 cells expressing low levels of Bim may be selected during Tax-induced transformation of CTLL-2 cells.

The second mechanism of Bim reduction in CTLL-2 cells is posttranscriptional reduction of Bim protein. Bim is known to be phosphorylated by Erk and degraded in a phosphorylation-dependent manner. Stoppa et al. showed that Tax expression in the human T-cell line Jurkat increased the levels of p-Erk, thereby inhibiting cisplatin-induced apoptosis [38]. They further showed that Tax activates Ras, an upstream activator of Erk, suggesting that Tax increases p-Erk by stimulating Ras activity. We also observed that Tax increased the amount of p-Erk present in CTLL-2 cells and that this activation was blocked by the MEK inhibitor U0126, suggesting Erk activation by Tax is mediated through the upstream kinase MEK. However, our data also demonstrated that Erk activation by Tax is not responsible for BimEL degradation. Since Tax has been reported to activate several kinases, including IKK*α* [39], IKK*β* [40, 41], AKT [42], mTOR [43], TAK1, and JNK [44], there are multiple candidates possibly responsible for BimEL phosphorylation by Tax. Although Bim degradation by Tax was unable to protect CTLL-2 cells from apoptosis after IL-2 withdrawal, such activity would have a critical role in establishing HTLV-1 infection in vivo.

In some HTLV-1 infected cell lines, Erk activation and BimEL phosphorylation did not correlate with the reduction of BimEL protein. It has been previously reported that Erk-dependent BimEL phosphorylation does not induce BimEL protein degradation in chronic lymphoblastic leukemia cells, but instead reduces its binding to the antiapoptotic protein Mcl1, thus contributing to cell survival [45]. It is possible that the same mechanism of BimEL inhibition operates in HTLV-1-infected cells or ATL cell lines.

Intriguingly, the amount of Bim protein was low in ATL-derived T-cell lines, even though they expressed a very small or nearly undetectable amount of Tax. Moreover, re-expression of Bim in ATL cell lines reduced cell growth by inducing apoptosis. These results indicate that the aberrant growth of ATL cells depends on Bim repression. Thus, Bim may be a good target molecule for therapeutics against ATL. For instance, a Bim mimetic, such as ABT-737, might be a good candidate for anti-ATL therapeutics [46].

In addition to Tax, several viral oncoproteins including LMP2A of Epstein–Barr virus and viral interferon regulatory factor 1 of human herpesvirus 8 inhibit Bim function [47, 48]. Specifically, LMP2A inhibits Bim-induced anoikis by promoting Erk-dependent Bim degradation in epithelial cells [47]. Thus, Bim is a common target for oncogenic viruses to establish persistent infection. Further investigations to elucidate the mechanism of Bim downregulation in HTLV-1-infected cells will advance the understanding of viral pathogenesis, including HTLV-1-induced ATL.
